# Twelve Tips for Students in Longitudinal Integrated Clerkships

**DOI:** 10.15694/mep.2019.000059.1

**Published:** 2019-03-19

**Authors:** Joshua Pepper, Nardine Saad Riegels, Tal Ann Ziv, Lindsay Mazotti

**Affiliations:** 1 University of California; 2 Kaiser Permanente; University of California

**Keywords:** Longitudinal integrated clerkships, LICs, undergraduate medical education, growth mindset, student-directed learning, codesign

## Abstract

This article was migrated. The article was marked as recommended.

Longitudinal integrated clerkships (LICs) are a curricular structure for medical clerkships grounded in continuity across learning environments and experiences. There has yet to be a peer-reviewed article directly advising students in LIC programs. Twelve tips were created based on a comprehensive literature review of LICs and supported by the cumulative experience of the authors. They are ordered in four sequential groups: The first three tips discuss the importance of the relationships that are built between students and their patients (Tip 1), preceptors (Tip 2), and peers (Tip 3). Next we cover health systems, and offer advice on how students can integrate their learning (Tip 4), use technology to their advantage (Tip 5), and practice systems thinking (Tip 6). We then discuss the educational benefits when students take an active role in patient care (Tip 7), their own learning (Tip 8), and the feedback process (Tip 9). Finally, we cover the importance of self-care (Tip 10), reflection (Tip 11) and patience (Tip 12) during a LIC. These tips are designed to help students understand the pedagogical theory that underpins LICs, take an active role in their education, and maximize learning and wellness during their clerkship.

## Introduction

The longitudinal integrated clerkship (LIC) is a model of clerkship training for medical students; as opposed to conventional “block-based” rotations, LICs are designed to provide continuity across learning environments and experiences. The LIC educational experience is therefore grounded in longitudinal relationships with preceptors, patients, peers, places, and pedagogy ( Hirsh *et al.*, 2007; Alliance for Clinical Education, 2016). In the prototypical structure, students are assigned one preceptor for each core discipline and train within one healthcare system over the course of a year in an apprentice-like paradigm ( Knerr, Personnaz and Dreyfus, 1990).

Adoption of LICs has spread in part due to calls for curricular reform in medical education, particularly the need to train physicians for modern healthcare delivery systems and incorporate professional identity formation into medical training ( Berwick and Finkelstein, 2010; Cooke, 2010; Hirsh and Worley, 2013; Lucey, 2013). As of March 2018 there are at least 98 longitudinal programs in both urban and rural settings throughout the United States, with approximately half of all medical schools nationwide implementing some form of longitudinal curriculum ( Gheihman *et al.*, 2018). LICs are also growing internationally, with programs in at least seven countries ( Worley *et al.*, 2016).

Literature on LICs has increased exponentially over the last decade. LICs have been shown to increase students’ long-term learning ( Dent, Harden and Hunt, 2017, chap. 12), patient-centeredness ( Gaufberg *et al.*, 2014), and clinical independence ( Hauer, Hirsh, *et al.*, 2012) as they revisit medical knowledge across a variety of contexts. LICs also help students build trusting relationships with their preceptors ( Hirsh, Holmboe and ten Cate, 2014; Latessa *et al.*, 2017) and develop meaningful roles in the care of patients ( Hirsh and Worley, 2013). Current literature also includes best practices on developing LICs ( Poncelet *et al.*, 2011; Ellaway *et al.*, 2013; Chou *et al.*, 2014). Despite these resources, and despite recently published articles aimed at guiding students in traditional clerkships ( Bharamgoudar and Sonsale, 2017), to our knowledge there has yet to be a peer-reviewed and literature-based resource directly aimed towards students to help contextualize the LIC learning experience and provide tools to guide their holistic success. This article seeks to address this gap.

These tips were developed iteratively by incorporating the available literature and the cumulative experience of the authors. Within each tip, we first address the pedagogical evidence and relevant learning theories before citing practical implementations for the LIC student. The tips are ordered in four sequential groups: The first three tips discuss the importance of the relationships that are built between students and their patients (Tip 1), preceptors (Tip 2), and peers (Tip 3). Next we cover health systems, and offer advice on how students can integrate their learning (Tip 4), use technology to their advantage (Tip 5), and practice systems thinking (Tip 6). We then discuss the educational benefits when students take an active role in patient care (Tip 7), their own learning (Tip 8), and the feedback process (Tip 9). Finally, we cover the importance of self-care (Tip 10), reflection (Tip 11) and patience (Tip 12) during a LIC.

## Tip 1: Let your patients be your teachers

In LICs, students often form close relationships with their patients ( Gaufberg *et al.*, 2017) and have the opportunity to care for them across the entire course of illnesses, rather than providing episodic care ( Ogur *et al.*, 2007). This dynamic has multiple benefits. First, continuity improves students’ understanding of the social and cultural context of illness and its personal impact on their patients ( Shapiro, 2008). Second, students develop a stronger sense of patient-centeredness ( Hirsh *et al.*, 2012), which persists into their professional careers ( Gaufberg *et al.*, 2014). Finally, it facilitates patient observation of the student’s growth as a clinician over time and provides valuable perspective and feedback. Interviews of patients who worked with students in a LIC reported feeling “empowered by being included in the healthcare team, partnering with the doctor to professionally ‘form’ the student” ( Hudson, Knight and Weston, 2012).

Seeing patients as “experts by experience” often requires a change in mindset on the part of students and preceptors ( Donaghy, Boylan and Loughrey, 2010; Towle *et al.*, 2014). However, there are several concrete ways to encourage this practice. Examples include:

• Ask yourself after each patient encounter, “What has this patient taught me?”
• Keep a journal of memorable moments with patients and refer to it periodically.
• Schedule time to talk with patients after an office visit or during a hospital stay to tell you the story of their healthcare journey. During these conversations, let them know that their perspective on their illness is critical. The hero’s journey model can help you contextualize patient narratives and explore common archetypes ( Lamprell and Braithwaite, 2016).
• Asking a simple open-ended question, such as “What is your greatest concern today?” ( Riegels *et al.*, 2018) cues the patient that you are listening to them.

## Tip 2: Invest in strong relationships with your preceptors

Unlike conventional clerkships, in which students may switch attendings as often as each week, ( Hirsh, Holmboe and ten Cate, 2014) longitudinal preceptors come to understand a student’s “skills, abilities, and learning needs” over the course of the year ( Hauer, Hirsh, *et al.*, 2012). This leads to a more collaborative, rather than hierarchical, dynamic ( Worley *et al.*, 2006; Hauer, Hirsh, *et al.*, 2012). Moreover, LIC preceptors are able to witness the development of clinical skills and confidence, and help students track what patients and illnesses they have seen together ( Dornan *et al.*, 2007; Hirsh *et al.*, 2007). Preceptors in LICs see trainees as “ *their* *student*” as opposed to just “ *a student*” ( Dent, Harden and Hunt, 2017). Ultimately, longitudinal relationships with preceptors leads to greater student autonomy, ( Hauer *et al.*, 2014; Latessa *et al.*, 2017) more effective feedback, ( Hirsh *et al.*, 2007; Latessa *et al.*, 2017) and enhanced clinical performance ( Hirsh *et al.*, 2007). Longitudinal precepting also leads to greater educator satisfaction ( Walters *et al.*, 2011).

This may explain why most students in LICs cite continuity with preceptors as a major contributing factor to their success ( Mihalynuk *et al.*, 2008; Latessa *et al.*, 2017). Students attest that this relationship provides a supportive and trusting environment in which they feel “safe to make mistakes” ( Hirsh, Holmboe and ten Cate, 2014; Latessa *et al.*, 2017). These trusting relationships with preceptors are essential for efficient learning, conflict resolution, and integrating students within a community of practice ( Dornan *et al.*, 2007; Walters *et al.*, 2011; Bates *et al.*, 2013; Zak, 2017; Wolfe, Hoang and Denniston, 2018).

Your relationships with preceptors will evolve throughout the year. In the beginning of your LIC, take time to get to know your preceptor and ask them for an orientation to their clinical practice. Inquire if they have a preferred style of precepting. Share your expectations and goals for the first several months of the year, and develop a shared mental model for your current and future roles in the clinic workflow. Find time during the year to share your career goals with your preceptor and experiences about your life outside of medicine. Remember that relationships drive learning, and connecting with your preceptor will facilitate enhanced feedback, accountability, and independence.

## Tip 3: Collaborate with your LIC peers

Students in LICs typically form cohesive small-group relationships, which offer support and enhance workplace learning ( Bandura, 1986; Ten Cate and Durning, 2007; Chou *et al.*, 2011, 2014; Woolf *et al.*, 2012; Masters, O’Brien and Chou, 2013). LIC student groups often augment their learning by sharing more cases with each other ( Teherani, Irby and Loeser, 2013). Peers also guide one another to effectively manage cohort patients, navigate the electronic health record, and perform physical exam skills and procedures ( Chou *et al.*, 2014).

There are many ways to enhance peer learning. Several example include:

• Ask your classmate to shadow you during clinic or to review a note and offer feedback.
• Discuss challenging cases in appropriate venues with your classmates.
• Take time in the evening or on weekends to gather with your peers outside of the learning environment. A group hike, picnic, or meal together will help foster resilience, trust, and peer respect. These events might include fellow students, members of the health care team, and community members.
• Seek out relationships with your LIC alumni. Learn from their wisdom on topics such as best practices, strategies to avoid common pitfalls, clinical opportunities, professional development, career planning and thoughts about transitioning from the LIC year to your next steps.

## Tip 4: Integrate learning topics across specialties

Caring for patients across multiple settings leads to a sense of “doctor-like” responsibility as students directly support patients during a “whole illness experience” and help communicate between medical teams. ( Ogur *et al.*, 2007; Hauer, Hirsh, *et al.*, 2012). Self-directed learning time, intentionally incorporated into most LIC curricula, provides an opportunity to cross disciplines ( Ogur *et al.*, 2007; Poncelet *et al.*, 2011). Students may also benefit from seeing their patients’ problems as learning themes that transcend multi-disciplinary silos ( Harden, 2000). Interleaving medical concepts across settings is thought to help students improve long-term memory ( Weinstein, Madan and Sumeracki, 2018), faculty and staff relations ( Dent, Harden and Hunt, 2017), inductive learning, and differentiation between similar problems types ( Birnbaum *et al.*, 2013).

The benefit of integration also extends to patients. Patients appreciate their LIC student functioning as a “bridge” between settings and providers, particularly when their condition is complex or they are chronically ill ( Poncelet *et al.*, 2013).

There are many ways you might play a role in integrating your patients’ care across specialties. Early in the year, consider using your student-directed learning time to orient to multiple inpatient and outpatient settings. This will prepare you to visit your patients in environments such as the emergency department or hospital. Work with your preceptor to identify patients who have complex care needs. Examples include (1) elderly patients, (2) patients with chronic pain, stroke, recent hospitalization, (3) patients with congenital conditions, autoimmune disease, or cancer, and (4) pregnant patients. Communicate with these patients and your providers about your role in the care team across departments.

## Tip 5: Use technology to your advantage

Students in LICs rely on technology to enhance their learning, communicate with teams, and track their cohort of patients throughout the year ( Alliance for Clinical Education, 2016, chap. 16; Dent, Harden and Hunt, 2017, chap. 20). While technologies are generally comparable across different clerkships, LIC students often use one health record system for the whole year. Some LIC programs have advanced systems for managing patient cohorts such as notifications when a patient is admitted or in the emergency department ( Poncelet *et al.*, 2011). Students in rural LIC programs may find technological tools particularly useful for staying connected to their preceptors, patients and their program across disparate departments and clinical sites ( Alliance for Clinical Education, 2016, chap. 16). Regardless of the technological systems available, students readily adapt to the learning cultures and contexts of their learning environments and often end up with an individualized approach to technology ( Ellaway *et al.*, 2014).

We recommend that you orient to the technologies available at your institution early and develop routines that optimize these tools in your clinical practice. For example, before an outpatient clinic session you may review patients’ histories in the electronic health record (EHR) and develop a list of relevant learning objectives. Use available online resources to research your learning objectives, add to your knowledge base, and test yourself on this material (see Tip 8). You may also choose to use podcasts, digital lectures, wikis, and online reference materials to enhance your learning on these subjects. Share useful resources with your peers and preceptors. Seek preceptor guidance to become facile with technologies in the clinic. Lastly, your post-clinic routine might include tracking cases you have seen in a digital logbook ( Schüttpelz-Brauns *et al.*, 2016) or portfolio to guide your learning.

## Tip 6: Practice systems thinking

Systems thinking refers to a focus on systems as the context for defining and solving problems ( Sweeney and Meadows, 2010). Systems thinking in healthcare contextualizes individual patient interactions in the underlying health care delivery system, behavioral and social determinants of health, population health, health care economics, and patient safety and quality improvement ( Hawkins *et al.*, 2016, p. 5). The LIC offers a unique platform from which to acquire systems thinking skills, in that students experience continuity of relationships with both patients and place ( Latessa *et al.*, 2017). This cultivates a nuanced understanding of healthcare delivery from the lens of the patient.

While various LIC programs have implemented specific curricula in quality improvement ( Alliance for Clinical Education, 2016, chap. 14), it is possible even without a structured curriculum to practice systems thinking in your daily work. The following practices might help you develop this skill:

• Seek to understand the big picture.
• Identify the circular nature of complex cause and effect relationships.
• Use an understanding of system structure to identify possible leverage actions.
• Change perspectives to increase understanding. ( Benson, 2015)

To develop systems thinking, practice these habits during patient encounters, handoffs, and transitions ( Alliance for Clinical Education, 2016, p. 124). If you see patterns of deficiencies at your clinical site, consider getting involved in a systems improvement project to address root causes. If you decide to obtain formal training in health systems or quality improvement, your patients’ stories and experiences will anchor your skills and allow you to apply them more purposefully.

## Tip 7: Take ownership of your patients’ care

LIC students are more likely to frame their clinical experience as “work”, reflecting a sense of ownership and vocation in their learning ( Worley *et al.*, 2006). Whereas block-based students are often expected to display the qualities of a performer, LIC students more regularly embody the qualities of a caregiver: patient-centeredness, autonomy, and responsibility ( Ogur and Hirsh, 2009; Hauer, Hirsh, *et al.*, 2012; Hauer, O’Brien, *et al.*, 2012; O’Brien *et al.*, 2016). These attitudes and expectations may result from the increased autonomy experienced by many LIC students ( Mihalynuk *et al.*, 2008; Teherani *et al.*, 2009; Teherani, Irby and Loeser, 2013).

This increased responsibility has educational benefits. Direct patient care increases development of professional identity, ( Hauer, Hirsh, *et al.*, 2012; Bates *et al.*, 2013) clinical competency, ( Dornan *et al.*, 2007) and sense of purpose ( Worley *et al.*, 2006). Social learning theories posit that student participation in the community of practice is essential to medical education ( Taylor and Hamdy, 2013).

Table 1 describes opportunities to take ownership over patients’ care, both to further learning and to promote increasing levels of responsibility over the course of the LIC.

**Table 1.** How to take ownership of your patient’s care

Opportunities	Examples
During an outpatient visit	• Develop a list of cohort patients to follow longitudinally; identify appropriate new cases that will support your learning. • Review the diagnostic, therapeutic, and follow-up plan with your patients at the end of each visit. Identify barriers to care. • Draft an after visit summary for the patient to take home. • Let the patient know when you will contact them in the future. • Try the SNAPPS method of ambulatory case presentations with your preceptor (Wolpaw, Wolpaw and Papp, 2003).
Between outpatient visits	• Set reminders to follow up with your patients. • Develop a system to review new laboratory or imaging results. • Consult with a pharmacist to resolve a medication question. • Reach out to a social worker for input about resources for a patient. • With permission, join a home visit or family meeting.
In the inpatient setting	• Spend time with your patients after rounds to revisit the plan and answer questions. • Help your patient formulate questions for the team before rounds. • Share and review educational resources for your patients and their families. • Offer to call your patient’s family members or friends to provide a medical update. • Ask your patients if you can call them after discharge or join them for outpatient follow-up appointments.

## Tip 8: Recall and test your knowledge over time

Students can express frustration when they find they must relearn a topic multiple times. However, LICs are intentionally structured to space learning throughout the year, ( Hirsh *et al.*, 2007) allowing students to “consolidate, reflect upon and even ‘necessarily forget’ learned material” ( Dent, Harden and Hunt, 2017, chap. 12). The effort students put into spaced retrieval leads to longer lasting learning ( Brown, Roediger and McDaniel, 2014).

There are many ways to actively recall and test your knowledge throughout a LIC. Ideal systems for tracking your knowledge are portable and can expand as your experience, knowledge and skills develop throughout the year and across clinical settings. You may choose to use an online note managing software, handwritten or printed notes, or a combination of these modalities. Digital flashcards and question banks are also strategies to test material and improve retention ( Deng and Gluckstein, 2014; Akresh-Gonzales, 2015), and can be developed as your knowledge bank grows. When returning to a particular specialty, take a few minutes to recall information you learned previously before re-reading your notes ( Brown, Roediger and McDaniel, 2014). You may also choose to build concept maps or tables comparing and contrasting cases you see throughout the year. No matter what methods you use to take notes and test your knowledge, try to incorporate your system into your pre-clinic, post-clinic and inpatient workflow so the material is contextualized and reinforced by the patients you see.

## Tip 9: Collect feedback and make learning goals

LICs preceptors observe students over many settings and patient presentations, providing a rich and essential opportunity for observation and feedback ( Ogur *et al.*, 2007; Poncelet *et al.*, 2011; Teherani, Irby and Loeser, 2013). Students and preceptors perceive feedback in LICs as more authentic, useful and constructive, possibly due to the structure of the clerkship and sense of safety ( Mazotti *et al.*, 2011; Bates *et al.*, 2013). The alliance of LIC preceptor and student may foster a fertile environment for actionable feedback and improved learning ( Telio, Ajjawi and Regehr, 2015).

We recommend that LIC students solicit feedback regularly. Ask your preceptor for feedback after any observed patient encounter and at the end of a clinic or an inpatient experience. Also seek feedback from other faculty or staff who interact with you longitudinally. Medical assistants, nurses, receptionists all observe your performance and may have important tips for you. Make note of recurring themes, as this may help identify high priority areas for improvement.

Then, process this feedback into learning goals. Some LIC students create individualized learning plans (ILPs), a “written contract” of learning goals. ILPs require a learner to identify their learning needs, find resources to meet those needs, and evaluate their achievement. ILPs also help document self-assessment and self-directed learning as part of the practice-based learning and improvement competency ( Shepard *et al.*, 2012; AcfGM, 2013). Be sure that your goals are important, specific, measurable, accountable, realistic and linked to a timeline for completion ( Conzemius and O’Neill, 2009). If you do not have a structured program to review learning plans, ask a clerkship director or faculty advisor to review your plans with you periodically.

## Tip 10: Practice self-care

Medical school has been associated with high rates of burnout, anxiety, depression and alcohol abuse ( Dyrbye, Thomas and Shanafelt, 2006; Dyrbye *et al.*, 2014; Jackson *et al.*, 2016). LICs themselves may promote resilience due their “secure, supportive learning environment” that mitigates the stress associated with clinical training ( Greenhill and Poncelet, 2013). Try to see your LIC cohort and program as a “safe haven” in which you might rejuvenate and debrief ( Greenhill *et al.*, 2015).

Develop your self-care practice. Exercising three times per week and nurturing relationships outside of medicine can be especially protective of mental health ( Noori, Sofia; Blood, Alyssa; Meleca, Joe; Kennedy, Vanessa; Sengupta, Debashree, 2017). Other examples of self-care include sleep, nutrition, hygiene, spiritual care, meditation and relaxation, time for loved ones, outside activities, and other hobbies. Students who disclose utilizing a multitude of self-care practices throughout their training may sustain greater resiliency and lower risk for distress during their medical education ( Ayala *et al.*, 2018). Creating habits of self-care will foster wellness and resiliency throughout your career.

Lastly, do not hesitate to seek support for anxiety, depression, feelings of isolation or suicidality or for substance abuse. Regrettably, these feeling remain more common among medical students than the general public, ( Goebert *et al.*, 2009) with over a quarter of medical students experiencing depressive symptoms and over ten percent endorsing suicidal ideation ( Rotenstein *et al.*, 2016). Many schools have created wellness curricula and resources including medical student well-being programs; these may be available through your Office of Student Affairs and resources abound online. If immediate resources are not available from your medical school, reach out to human resources or the Employee Assistance Program at your clinical site or online.

## Tip 11: Reflect on your experiences

Critical reflection on your clinical experiences is essential to student learning and professional development. Reflection can be accomplished through questioning, analysis, and reframing ( Aronson, 2011); we provide practical examples below. Such reflection has three main purposes.

First, reflection serves as a healthy coping strategy that builds resilience and prevents burnout ( Greenhill *et al.*, 2015). LICs provide a community in which to process logistical and emotional challenges, ( Treadway and Chatterjee, 2011) explore professional identity, ( Wald, 2015) and reaffirm your reasons for pursuing medicine ( Greenhill *et al.*, 2015).

Reflection is also a key component of service learning, ( Bringle and Hatcher, 1999) which is associated with improved teamwork, leadership, understanding of diversity, academic knowledge and professional skills ( Stewart and Wubbena, 2015).

Lastly, critical reflection promotes metacognition, ( Tanner, 2012; Colbert *et al.*, 2015; Medina, Castleberry and Persky, 2017) which is thought to reduce medical errors, improve study skills, enhance self-directed learning, promote critical thinking, ( Medina, Castleberry and Persky, 2017) and reduce cognitive bias ( Croskerry, 2003). Indeed, it has been posited that experience alone is insufficient to learn clinical medicine, and critical reflection is the “element that turns experience into learning” ( Arseneau, 1995).

We suggest you find a reflective practice that works for you and build it into your daily or weekly routine. Examples of a reflective practice include journaling, phone calls with a classmate, and regular meetings with a mentor. Suggested prompts for reflections include:

• What went well, what didn’t, and what will I do differently next time?
• What is one thing I learned and one thing I am still confused about? ( Tanner, 2012)
• What were my goals for the week, and did I accomplish them?
• What surprised me or delighted me about today? ( Hart, 2018)

Some LIC programs hold dedicated reflection sessions. Come prepared with specific stories of challenging cases, ethical dilemmas, issues of professionalism, and other topics you would like to share with your peers. We recommend taking notes about specific experiences with patients and healthcare systems during the year. Not only will these stories inform your reflection sessions, you may continue learning from them throughout your career.

## Tip 12: Trust the process

Longitudinal clerkships are designed for the specific outcome of training medical students to approach patients with a holistic, longitudinal and transdisciplinary lens. But this skill is attained gradually and can be elusive at times: a qualitative analysis characterized students’ stages of progression through a LIC as first transitioning to clinical learning, second “dealing with disorientation and restoring balance,” and finally identifying as a physician. ( Dubé *et al*., 2015).

In the first half of the year, LIC students may fear that they may not achieve mastery in individual disciplines, compared to peers in block clerkship who have had the opportunity to focus on one discipline at a time. However, students in LICs have been shown to have a gradual and sustained increase in fund of knowledge over the course of the year and better retention of knowledge compared to students in block clerkships ( Hansen and Simanton, 2009). Similarly, students in LICs describe progressive independence during the clerkship year, while their peers in block clerkships do not ( Hauer, Hirsh, *et al*., 2012).

These studies and our experience affirm that the unique structure of a LIC promotes an integrative and longitudinal learning process that may at first seem foreign to students. Speaking to near-peer students who have completed the program in the year or two before you can be helpful to calibrate expectations and gain perspective on the outcomes of the clerkship. If you feel concerned about your progress, seek out advice and input from preceptors or clerkship directors, whose experience can help you put your concerns into context.

## Conclusion

LICs are a novel approach to clinical undergraduate medical education that present a unique set of challenges to student learners. This paper highlights several key pedagogical themes for the student learner in facing these challenges.

First, LICs require students to see themselves as adult learners who prioritize their own time and effort. This manifests as proactively caring for patients (Tip 7), developing proficiency with available technology (Tip 5), finding opportunities to integrate learning across settings and cases (Tip 4), and tracking and testing their learning over time (Tip 8). Such self-directed learning ( Knowles, 1975) allows students to match their clinical exposure to their learning goals and participate meaningfully in the care of patients.

Second, these tips will also help students maintain a growth mindset ( Dweck, 2008). Medical students in block-based clerkships most often develop performance goals that are aimed at demonstrating competence and avoiding negative judgement ( Bates *et al.*, 2013). In contrast, we encourage LIC students to see their learning as malleable ( Dweck, 1990) and establish learning goals that reflect *learning orientation* rather than *performance orientation.* Practically, this is accomplished by developing trusting relationships with patients (Tips 1), preceptors (Tip 2), and peers (Tip 3), seeking out and incorporating longitudinal feedback (Tip 9), and critically reflecting on experiences (Tip 11).

Third, these tips are intended to help student learners maintain perspective on their learning. By learning about health systems, LIC students can learn to function within a healthcare team and understand structural forces on their patients’ health (Tip 6). Perspective also helps students maintain balance during a stressful year (Tip 10) and be patient with the arc of learning that occurs during a LIC (Tip 12).

The tips discussed in this paper will serve as scaffolding to succeed in your clerkship over time. As you gain knowledge, experience, and professional skills over the course of the year, you will add value to your patients ( Bernstein *et al.*, 2018), your training environment ( Hauer, Hirsh, *et al.*, 2012) and your education ( Levett-Jones *et al.*, 2007). This will allow you to integrate within a community of practice as you develop a sense of value, meaning and belonging in your work ( Li *et al.*, 2009).

## Take Home Messages

• Relationships with patients, preceptors, and peers in longitudinal integrated clerkships (LICs) drive students’ learning.
• Key skills in longitudinal clerkships for students include integrating learning across specialties, leveraging technology, and practicing systems thinking.
• Students can enact practical strategies to promote self-directed learning, enhance direct patient care and incorporate feedback.
• A longitudinal curricular structure provides opportunities for students to build skills of self-care, reflection, and patience.
• As students progress through an LIC, they gain independence in their learning and clinical practice, develop a growth mindset, and maintain perspective on their learning within the context of health systems and their own educational journeys.

## Notes On Contributors

Joshua Pepper is a fifth-year student in the UC Berkeley, UC San Francisco Joint Medical Program.

Nardine Saad Riegels is an Associate Professor of Clinical Medicine, University of California San Francisco and Associate Professor of Health Systems Sciences, Kaiser Permanente School of Medicine. She serves as Clerkship Director for the Kaiser Permanente-UCSF Longitudinal Integrated Clerkship, East Bay and Director of Quality Programs for Graduate Medical Education at Kaiser Permanente East Bay.

Tal Ann Ziv is an Associate Professor of Clinical Medicine, University of California San Francisco. She serves as Associate Director for the Kaiser Permanente-UCSF Longitudinal Integrated Clerkship, East Bay and Site Director, Internal Medicine Clerkship, University of California San Francisco, Kaiser Permanente East Bay.

Lindsay Mazotti is an Associate Professor of Clinical Medicine, University of California San Francisco and Associate Professor of Clinical Sciences, Kaiser Permanente School of Medicine. She serves as Assistant Physician in Chief for Education and Development at Kaiser Permanente East Bay and Director of Clinical Experience for the Kaiser Permanente School of Medicine.
