# Supplementary material for: Twelve Tips for Students in Longitudinal Integrated Clerkships
Source: MedEdPublish (2016). 2019 Mar 19;8:59. [Version 1] doi: 10.15694/mep.2019.000059.1 (PMC10712610; doi:10.15694/mep.2019.000059.1)
Supplement: Peer review history

# Reviewer report: Reviewer response for version 1

**DOI:** 10.21956/mep.19793.r29762

**Published:** 2019-04-05

**Authors:** Robyn Latessa

**Affiliations:** 1 UNC School of Medicine

This review has been migrated. The reviewer awarded 4 stars out of 5 This article addresses a gap in the literature regarding preparing an LIC student to maximize their experience. Though there is extensive research about LICs, there are few peer-reviewed articles specifically geared toward a student audience. The authors incorporate practical tips with comprehensive literature in a seamless manner. While some of the tips can be applied to any student entering their clinical rotations, much of the advice given is specific to the distinct aspects of LICs.I will plan to use this in our LIC program during orientation and will also share with other LIC programs.

Reviewer Expertise:

NA

No decision status is available

# Reviewer report: Reviewer response for version 1

**DOI:** 10.21956/mep.19793.r29765

**Published:** 2019-03-24

**Authors:** Nora Osman

**Affiliations:** 1 Harvard Medical School

This review has been migrated. The reviewer awarded 4 stars out of 5 In contrast to traditional block clerkships, longitudinal integrated clerkships (LIC) provide continuity of relationships and learning environment. LICs have demonstrable benefits to learner, preceptor, patient and community. There is a growing literature on LICs, and this manuscript adds to the literature, by elegantly describing the ways that students can be agents in their learning. The grouping of the tips makes sense. The references are extensive and create a valuable bibliography for any and all interested in the topic. What stands out most, however, is that all of the tips could be and should be used by students in all clerkships, longitudinal or otherwise. It is a very well written manual for all students entering the clinical learning environment.

Reviewer Expertise:

NA

No decision status is available

# Reviewer report: Reviewer response for version 1

**DOI:** 10.21956/mep.19793.r29761

**Published:** 2019-03-21

**Authors:** P Ravi Shankar

**Affiliations:** 1 American International Medical University

This review has been migrated. The reviewer awarded 4 stars out of 5 Longitudinal integrated clerkships provide a continuity of learning experiences and student feel more involved in the care of the patients. The twelve tips provided by the authors are grouped into those connected with relationships, health systems, educational benefits, and self-care, reflection and patience. With each tip the authors extensively cite the scientific literature before they provide practical advice to the student. The paper is well-written and makes for interesting reading. Longitudinal clerkships are becoming commoner and students and faculty members will find this article to be of interest.

Reviewer Expertise:

NA

No decision status is available

# Reviewer report: Reviewer response for version 1

**DOI:** 10.21956/mep.19793.r29763

**Published:** 2019-03-20

**Authors:** Trevor Gibbs

**Affiliations:** 1 AMEE

This review has been migrated. The reviewer awarded 4 stars out of 5 Although a rather long paper to read, I enjoyed reading this paper and found the reference very extensive and useful. We are still learning about the true value of LICs but this paper covers the more practical approach to their construction. I particularly thought that the student and patient-centredness of the authors' twelve tips were a welcome approach to the subject. I would encourage all those involved with LICs to read this informative and practical paper.

Reviewer Expertise:

NA

No decision status is available

# Reviewer report: Reviewer response for version 1

**DOI:** 10.21956/mep.19793.r29764

**Published:** 2019-03-20

**Authors:** Lee HangFu

**Affiliations:** 1 Windsor University School of Medicine

This review has been migrated. The reviewer awarded 2 stars out of 5 The title "Twelve Tips for Students in Longitudinal Integrated Clerkships" raised my interest because of the LIC program. The paper focused on the common learning experience that a student may face in an LIC core-rotation. The paper is a good introduction to LIC for novice students entering their Clinical experience. It lacks the value and integrated learning advantages compare to the block-based clerkship.

Reviewer Expertise:

NA

No decision status is available
